# The need for a network to establish and validate predictive biomarkers in cancer immunotherapy

**DOI:** 10.1186/s12967-017-1325-2

**Published:** 2017-11-03

**Authors:** Giuseppe V. Masucci, Alessandra Cesano, Alexander Eggermont, Bernard A. Fox, Ena Wang, Francesco M. Marincola, Gennaro Ciliberto, Kevin Dobbin, Igor Puzanov, Janis Taube, Jennifer Wargo, Lisa H. Butterfield, Lisa Villabona, Magdalena Thurin, Michael A. Postow, Paul M. Sondel, Sandra Demaria, Sanjiv Agarwala, Paolo A. Ascierto

**Affiliations:** 1 Department of Oncology-Pathology, Karolinska Institutet, 171 76 Stockholm, Sweden; 2 NanoString, Inc, 530 Fairview Avenue N, Seattle, WA 98109 USA; 3 Gustave Roussy Cancer Institute, 114 Rue Edouard Vaillant, Villejuif/Paris-Sud, France; 4 Robert W. Franz Cancer Center, Earle A. Chiles Research Institute, Providence Portland Medical Center, 4805 NE Glisan, Portland, OR 97213 USA; 5 Research Branch, Division Chief of Translational Medicine at Sidra Medical and Research Center, Doha, Qatar; 6 Abbvie Corp. Redwood City, California, IL 94063 USA; 7 IRCCS IRCCS Istituto Nazionale Tumori Regina Elena, via Elio Chianesi 53, Rome, Italy; 8 Department of Epidemiology and Biostatistics, College of Public Health, The University of Georgia, 101 Buck Road, Athens, GA 30602 USA; 9 Melanoma Section Department of Medicine, CSC Building Roswell Park Cancer Institute, Elm & Carlton Streets, 915, Buffalo, NY 14263 USA; 10 Department of Dermatology, Johns Hopkins University SOM, 600N. Wolfe Street Blalock 907, Baltimore, MD 21287 USA; 11 Department of Surgical Oncology, Division of Surgery, The University of Texas MD Anderson Cancer Center, Houston, TX USA; 12 Department of Medicine, Surgery and Immunology, University of Pittsburgh Cancer Institute, 5117 Centre Avenue, Pittsburgh, PA 15213 USA; 13 National Cancer Institute, Cancer Diagnosis Program, DCTD, National Institutes of Health, 9609 Medical Center Drive, Bethesda, MD 20892 USA; 14 Melanoma and Immunotherapy Oncology Service, Memorial Sloan Kettering Cancer Center, New York, NY USA; 15 Weill Cornell Medical College, New York, NY USA; 16 Sarcoma Oncology Service, Memorial Sloan-Kettering Cancer Center, New York, NY USA; 17 Departments of Pediatrics, Human Oncology and Genetics, University of Wisconsin, 4159 WIMR Building, 1111 Highland Ave Madison, WI USA; 18 Department of Radiation Oncology and Pathology, Weill Cornell Medicine, 1300 York Avenue, Box 169, New York, NY 10065 USA; 19 St. Luke’s University Hospital and Temple University, Bethlehem, USA; 20 Melanoma. Cancer Immunotherapy and Innovative Therapy Unit, Istituto Nazionale Tumori Fondazione “G. Pascale”, Via Mariano Semmola, 80131 Naples, Italy

## Abstract

Immunotherapies have emerged as one of the most promising approaches to treat patients with cancer. Recently, the entire medical oncology field has been revolutionized by the introduction of immune checkpoints inhibitors. Despite success in a variety of malignancies, responses typically only occur in a small percentage of patients for any given histology or treatment regimen. There are also concerns that immunotherapies are associated with immune-related toxicity as well as high costs. As such, identifying biomarkers to determine which patients are likely to derive clinical benefit from which immunotherapy and/or be susceptible to adverse side effects is a compelling clinical and social need. In addition, with several new immunotherapy agents in different phases of development, and approved therapeutics being tested in combination with a variety of different standard of care treatments, there is a requirement to stratify patients and select the most appropriate population in which to assess clinical efficacy. The opportunity to design parallel biomarkers studies that are integrated within key randomized clinical trials could be the ideal solution. Sample collection (fresh and/or archival tissue, PBMC, serum, plasma, stool, etc.) at specific points of treatment is important for evaluating possible biomarkers and studying the mechanisms of responsiveness, resistance, toxicity and relapse. This white paper proposes the creation of a network to facilitate the sharing and coordinating of samples from clinical trials to enable more in-depth analyses of correlative biomarkers than is currently possible and to assess the feasibilities, logistics, and collated interests. We propose a high standard of sample collection and storage as well as exchange of samples and knowledge through collaboration, and envisage how this could move forward using banked samples from completed studies together with prospective planning for ongoing and future clinical trials.

## Introduction

### Concept 1. The need for biomarker discovery and validation in cancer immunotherapy

Immunotherapies have emerged as one of the most promising approaches to treat patients with cancer. Recently, the entire medical oncology field has been revolutionized by the introduction of immune checkpoint inhibitors, including T cell inhibitory receptors such as cytotoxic T-lymphocyte-associated antigen 4 (CTLA-4) and programmed cell death-1 (PD-1) or its ligand (PDL-1). However, despite well documented success in a variety of malignancies, responses typically only occur in a small percentage of patients for any given histology or treatment regimen. There are also concerns associated with immune-related toxicity and the high cost of immunotherapies. Because of this, identifying biomarkers to determine those patients that are most likely to derive clinical benefit from different immunotherapies and those who are more prone to develop adverse side effects is a compelling clinical and social need. Moreover, with several new immunotherapy agents in different phases of development, and approved therapies being evaluated in various combinations with different standard of care treatments, there is an urgent requirement to stratify patients and select the most appropriate populations in which to assess clinical efficacy.

Because of the complexity of the immune response, tumor heterogeneity and patient diversity, it is unlikely that a single biomarker will be sufficient to predict clinical outcomes in response to the spectrum of immune-targeted therapies. Biomarkers which are correlated with clinical outcome can be identified at molecular (genetics, epigenetics, metagenomics, proteomic, metabolomics, etc.), cellular and tissue levels. Before a candidate biomarker and/or new technology can be used for treatment decisions in a clinical setting, several steps are necessary to demonstrate its clinical validity. The discovery and assessment of biomarkers using cutting edge technologies across different clinical studies is a fundamental step in maximizing data generation. Collaborative efforts to combine clinical trial samples and data will empower data analysis and the significance of any biomarkers identified.

A biomarker with clinical relevance requires rigorous validation which can be separated into several sequential steps: assessment of basic assay performance (analytical validation); characterization of the assay performance with regard to its intended use (clinical validation); validation in clinical trials that ensures that the assay performs robustly according to predefined specifications (fit-for-purpose) and the establishment of definitive acceptance criteria for clinical use (validation of clinical utility). The fit-for purpose approach (an umbrella term used to describe distinct stages of the validation process) for biomarker development and validation addresses the proper assay tailored to meet the intended purpose of the biomarker. The Society for Immunotherapy of Cancer (SITC) Immune Biomarkers Task Force convened to address this need in this two-volume series; pre-analytical and analytical (Volume I) as well as clinical and regulatory (Volume II) aspects of the validation process as applied to predictive biomarkers for cancer immunotherapy [1, 2].

Clinical study design in which biomarker analysis is one of the primary objectives/endpoints needs to be promoted. A good example of such a study was CA184-004 (NCT00261365), a phase II trial to determine predictive markers of response to ipilimumab (MDX-010). In this study, the primary endpoint was to identify candidate markers predictive of response and/or serious toxicity to ipilimumab. Tissue and blood samples were collected at different time points from enrolled patients and the subsequent biomarker analyses generated interesting data. The findings of this study could have been considered as the first evidence for biomarker association with outcome, and could potentially have been confirmed and prospectively validated in subsequent studies. However, study results can often not sufficiently meet expectations or may be contradictory, which may be in part due to the constraints of underpowered cohort size, variables in the time and type of sample collection, and differences in procedures, data generation and tools used at different sites. The opportunity to design parallel biomarker studies that are integrated as key components of important randomized clinical trials could offer a solution. Sample collection (fresh and/or archival tissue, PBMC, serum, plasma, stool, etc.) at specific stages of treatment is important to evaluate possible biomarkers and to study mechanisms of response, resistance, toxicity and relapse. Such studies would be helpful in the design and development of upcoming therapies. At present, several research institutes around the world, including many at which the authors of this article are based, are collecting tumor, blood, serum and faecal samples to investigate prognostic and predictive biomarkers and to better understand the complex immunobiology of patients and their cancers.

Biomarker discovery is a fundamental objective in the design of many clinical trials. Therefore, the incorporation of correlative biomarker studies using state-of-the-art technologies within clinical trials in order to maximize data generation is required. The challenge at this stage is that most completed or ongoing clinical trials have not sufficiently incorporated biomarker assessment into their design. There is a need for an international joint effort to maximize data, information and knowledge generation from existing and completed clinical trials and to design clinical trials that will better address these important issues.

The proposal of this white paper is to encourage the creation of a network to facilitate the sharing and coordinating of samples from clinical trials in order to allow more in-depth analyses of correlative biomarkers than is currently possible. The feasibility, logistics, and various stakeholder interests in such a network are also considered. A high standard of sample collection and storage as well as the exchange of samples and knowledge through collaboration is proposed, and we envisage how this could move forward with banked samples from completed studies as well as with the prospective planning of ongoing and future clinical trials.

### Concept 2. The proposal of an international cancer immunotherapy biomarker consortium

There are a number of compelling arguments for the establishment of an international cancer immunotherapy biomarker consortium, which are summarized below.

1. To maximize the potential of novel biomarker discovery using samples from multi-institutional clinical practice and clinical studies and will offer a new breadth of experience and expertise to biomarker studies.
2. To allow streamlined sample access by developing online registration, biobanking, and the inventory and tracking of archived samples in order to best utilize samples existing after designated trials completed.
3. To set up and share standard operating procedures (SOPs) to harmonize future sample collection processing and banking.
4. To improve access to samples by initiating a new patient sample registry or by joining forces with existing international clinical trial patient registries with available clinical data and biological sample collection, including storage conditions and inventory information.
5. To provide support/guidelines in correlative study design.
6. To engage and leverage with cancer societies, pharma and biotech companies, and government institutes in order to improve the development of biomarkers.
7. To accelerate biomarker development by bringing together groups from around the world that can collaborate from proof-of-concept to validation. Moreover, the experience of the immunoscore worldwide project, coordinated by SITC, is an important example of an ‘honest broker’ approach for coordinating a specific study and data sharing [3].
8. To reinforce the concept of a stable-standing consortium which would be able to take a short, intermediate and long-term view towards biomarker development for more effective care of patients with cancer.

In order to emphasize the point 4# proposal above and to have data on as large a group of patients as possible, it would be very beneficial to have a stable-standing consortium among the major contributing institutions, societies and research centers. Recently, a consortium organized by The University of Tubingen was able to collect samples from several different institutions and obtain results from a large number of patients treated with Ipilimumab or an anti-PD-1 (Nivolumab or Pembrolizumab) [4]. The worldwide Immunoscore project is another good example of a effective consortium that was successful at validating a previously described biomarker [3].

### Concept 3. The challenges in biomarker discovery

A series of challenges are confronted when a project of this magnitude is proposed:

1. Limited or fragmented resources.
2. Insufficient numbers of patients per cohort.
3. Inclusion of patients with diverse treatment history (previous antitumoral treatment), histological and radiographic conditions.
4. Limited and/or suboptimal correlative study design due to funding and/or regulatory constraints.
5. Heterogeneity in the types of biological samples with different time points of collection, storage conditions, platforms used for data generation, lab-driven SOPs and data analysis algorithms and tools.
6. Heterogeneity in the clinical data collected and the length of follow-up,
7. Lack of international joint initiatives.
8. Potential intellectual property.

Previous programs that utilized treatment stratification biomarkers had higher success rates at each phase of development versus the overall dataset [5]. Moreover, the importance of determining biomarkers for both patient selection for treatment and selection of treatment for the patient is of utmost importance. As an example, assessment of tumor PD-L1 status is not critical for selecting patients with metastatic melanoma for treatment with anti-PD-1 inhibitors (Pembrolizumab or Nivolumab). In fact, patients with PD-L1 negative tumors may receive long-term benefit from anti-PD-1/PD-L1 treatment. However, PD-L1 status might be important for selecting patients for combination treatment (e.g. anti-CTLA-4 and anti-PD-1). Currently available data show that overall survival (OS) in PD-L1 positive patients treated with combined anti-CTLA-4 and anti-PD-1 is not superior to nivolumab monotherapy. This finding raises the question of why should these patients be treated with a more toxic regimen if the same benefit is achieved with less toxic monotherapy? However, PD-L1 expression is not the best example of a biomarker for patient selection in the context of checkpoint inhibitors. Although patients with strongly positive (> 50% on tumor cells) PD-L1 tumors clearly had an advantage for anti-PD-1 therapy versus chemotherapy in the Keynote 024 study, it is less clear why the same patient population did not show the same advantage from nivolumab therapy in the CheckMate 026 pivotal study [6]. Anti-PD-1/PD-L1 treatments showed similar efficacy across different clinical trials and the problem may be in the IHC assay and the ‘immunological’ characterization/criteria used to evaluate PD-L1 positivity. However, the FDA recently (11th May 2017) approved the combination of chemotherapy with pembrolizumab for first-line treatment of NSCLC regardless of PD-L1 expression, confirming the need for predictive biomarkers [7].

Over 10 years ago, the FDA issued important guidance that all drugs should be accompanied by a companion diagnostic (CDx). Industry has applied a thorough understanding of biology and the immune system to develop robust and meaningful biomarkers. While industry may believe that leveraging biomarkers and companion diagnostics are critical to precision medicine, developing them is challenging and expensive and therefore has been less of a priority. On the other hand, the introduction of Pembrolizumab in clinical trials should be an example to others. Industry efforts might be helped by strategic partnerships with academic institutions in order to identify relevant clinical biomarkers. For example, the processes of detecting reproducible, predictive biomarkers and developing robust companion diagnostics substantially benefit from the correlation to genomics and complex tissue analysis data. This includes the analysis of spatial relationships between immune cells hosting the tumour environment and the development of highly sensitive, precise, quantifiable and reproducible assays. These issues are challenging, time-consuming and need large investment. Pursuing a thorough, evidence-based and scientifically-driven drug discovery and development program is the desired long-term goal. However, funds to support these programs could be used for more short-term plans such as recruiting additional patient groups into a clinical trial in order to meet regulatory objectives.

At present, the best example of a truly successful and important immune-oncology predictive biomarker is mismatch repair deficiency: Cancer patients with microsatellite instability high (MSI-H) tumors independent of the tissue of origin benefit from immunotherapy with PD-1 inhibitors [8]. In particular, in colorectal cancer, MSI-H patients experience up to 50% or higher responses, while few if any responses are seen in MSI-low (L) colorectal cancer patients. Clinical demonstration of this concept has led to a recent approval by the FDA. We believe that these two examples of single predictive biomarkers (i.e. PD-L1 and MSI) will be the exception and not the rule and that we will have to search for the integration of several different biomarkers. This will amplify the challenge and will require a highly coordinated effort across multiple institutions.

### Patient inclusion and statistical considerations according to the study design

**Fig. 1:**
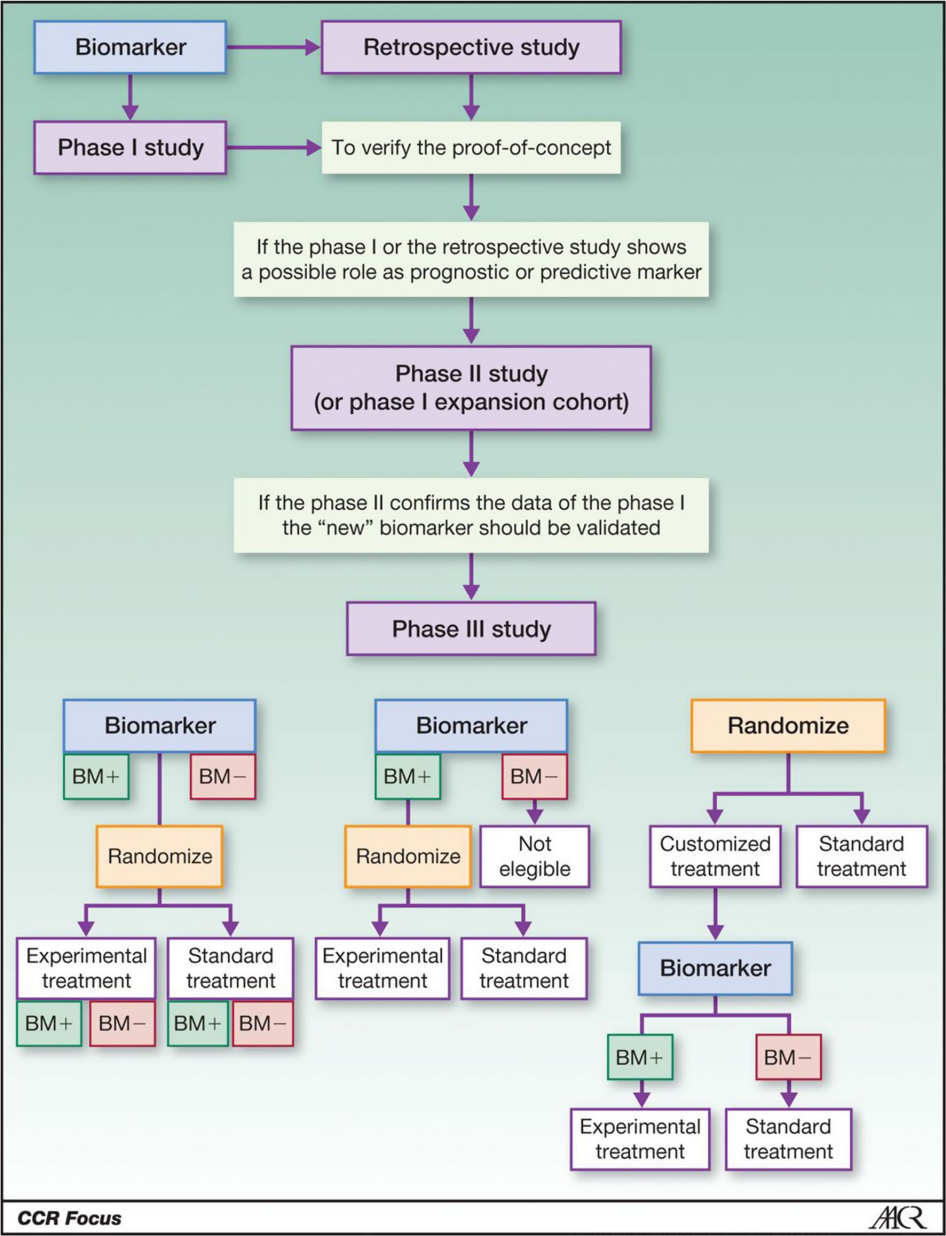
Procedures for discovery and validation of biomarkers. Alternative designs of randomized phase III trials in the presence of a potentially predictive marker of efficacy of treatment. BM + , positive biomarker; BM − , biomarker negative. Bottom left, “randomize-all” design with determination and prospective stratification of BM + and BM − patients. Center, “targeted” design. Right, “customized” design

The development and implementation of appropriate biomarker assays to study T cells and other cells in the microenvironment is an essential companion objective for clinical trials that seek to evaluate immunotherapeutic agents, particularly when used in combination. In principle, the efficacy of a compound (in this case immune-targeted agents) is at least partially dependent on the presence of the target in the tumor. In an ideal scenario, when complete information on predictive factors and proper selection of patients can be obtained in the early phases of drug development (phase I–II studies), the conduct of subsequent phase III studies could be optimized. Unfortunately, this ideal scenario rarely occurs. The clinical immune-oncology research community is dealing with several key questions, including; (a) what metrics are best for biomarker evaluation in phase III studies, (b) what primary and secondary endpoints should ne assessed, and (c) what are the statistical properties of various metrics. When planning a phase III trial comparing an experimental treatment with the standard, we often have evidence supporting a predictive role of a biomarker, whereas patients with the absence of such expression should not respond. In such a scenario, different strategies are theoretically possible (see Fig. 1): (i) a ‘randomize all’ strategy, i.e. randomization between standard and experimental treatment without selection, possibly with stratification based on biomarker status (in this case, ‘stratified trial design’ or ‘treatment-marker interaction design’); (ii) ‘targeted’ design, i.e. randomization between standard and experimental treatment only in patients selected according to the status of the marker (also called ‘enrichment design’); and (iii) ‘customized’ strategy (also called ‘marker-based strategy’), i.e. randomization between a standard arm in which the treatment is the same for all patients, and a personalized arm in which treatment is chosen on the basis of the marker status of each patient.

How can we develop a predictive biomarker in a more efficient way? The experience of the worldwide consortium on Immunoscore could be leading the way in finding different trial methods for evaluating immune-oncology biomarkers. We could also consider an opposite approach to the classical flow suggested in Fig. 1 in which biomarkers are validated first. The challenge in this case is in the ability of statistics to find the correct solutions and avoid decades-long clinical trials. Since biomarkers have been validated preclinically they could be used in different cohort studies for clinical predictive analysis. This could immediately eliminate the lack of effective information in those markers that do not reach the desired endpoint.

## Methods

To address these challenges, a systematic approach involving a multi-institutional effort could help identify and develop robust, standardized biomarkers and related clinical data that support the selection and testing of promising therapeutic approaches and combinations. The diversity of reagents and platforms that are being used to assess the immune systems of humans and data reporting procedures are not consistent. This hampers data reproducibility among laboratories, which may prevent meaningful interpretations across studies and could lead to selection of different intent-to-treat (ITT) populations. Most of the assays being used involve high-throughput multi-parametric ‘signatures’ that require considerable statistical and bioinformatic efforts for proper algorithm development and robust data interpretation. The requisite capabilities are not available to all investigators assaying immune biomarkers and are not consistently being accomplished in academic or clinical laboratories due to resource constraints. This issue must be addressed to ensure that biomarker measurements using high-quality, validated assays can be compared between clinical trials. The use of different assays, if there are standards or reference materials available, could allow for evaluation and comparison of performance across the assays and across different sites. There is no current system that can easily integrate analyses across different clinical trials.

Different approaches to overcome these limitations and to address different technical and logistical challenges have evolved in the process of biomarker standardization.

- A central laboratory approach is able to provide integrated testing, sample management, and data-management services; therefore, it may be able to supply efficient and reliable biomarker testing and data delivery as part of comprehensive biomarker characterization. A small, integrated network of laboratories with specific expertise in an array of molecular and cellular approaches, testing blood and tumor samples, would be able to support coordinated and standardized biomarker investigation, sample sharing and data sharing.
- An alternative approach that facilitates the comparability and integration of data across multiple laboratories is assay harmonization (proficiency panel-driven SOPs, approaches and troubleshooting). Harmonization of biomarker assays enforces identical reagents and/or protocols across laboratories and the establishment of assay-specific protocols in individual laboratories. The harmonization process involves the participation of multiple laboratories in a consortium-based iterative testing process to identify the variables crucial for assay performance, with data sharing and sometimes centralized analysis. The ‘Immunoscore’ validation initiative is an example of this approach.
- An example that addresses comparability of approach across multiple IHC-based PD-L1 tests was addressed by the Blueprint Project developed by four pharmaceutical companies (Bristol-Myers Squibb, Merck & Co. Inc., AstraZeneca PLC, and Genentech, Inc.) [9].

## The network

Samples associated with clinical outcomes from clinical practice or clinical studies are an enormous resource for the identification of biomarkers that is often underutilized. We propose developing an International Immunotherapy Biomarkers Consortium to overcome barriers to the use of these samples and associated clinical information and to optimize the information that can be learned from them. This can help the development of biomarkers from proof-of-concept to validation. Moreover, the experience of the Immunoscore worldwide project coordinated by SITC is an important example of how to work in this field. The network should be a permanent consortium with a virtual biobank that can be accessed as soon as an investigator from the Consortium has a scientifically valid idea. Suggestions for such a consortium include:

1. A survey to establish the need and willingness of different institutions to participate with time, reagents, and sample sets. This would involve development of a subcommittee to explore issues and strategies for sharing licensing and royalty income for IPs developed from the consortium with centers who participate in providing tissue samples and scientific expertise.
2. A database and registry of ongoing international clinical trials; clinical information including outcome; survey of sample type and availability; clinical and experimental data have already been completed [4]. This exercise will help identify gaps, unify standards and understand the willingness of participants.
3. A committee to propose overall direction and identify projects to pursue, based on data-based information and sample availability. Other responsibilities of this committee would include:
  - Announcing survey results and providing database access to all participants.
  - Requesting proposals from participants and the broader community.
  - Seeking sponsorship from venture capital, pharma, society, and private funding (with the possibility to have exclusive rights to develop companion diagnostics).
  - Review proposals and grant-selected projects with sample access for phase I discovery up to phase III validation studies. The consortium will develop policies and procedures to ensure that samples will be used in the most effective, ethical way and to maximize data generation and knowledge generation using state-of-the-art methodology germline and somatic genetic analysis (SNP, NGS, CNV, TCR).
    i. Epigenetics (transcription, methylation, miRNA, splicing variation, fusion transcripts, transcription regulation).
    ii. Proteomics (serum/plasma protein profiling, cytokine assays, antibody screen, exosome analysis,
    iii. Metabolomic profiling.
    iv. Microbiome/virome analysis.
    v. Flow cytometry, Single cell network analysis, sorted cells, functional and phenotypic profiling.
    vi. Evaluation of the tumor and relationship of immune cells with image analysis and machine learning (Multiplex IHC, Immunoscore, CyTOF).
    vii. Pharmacogenomics.
    viii. ImmunoPET.
  - Monitor project progression and outcome.
  - Present updates to the consortium at international meetings.
  - Publish results of discovery as a consortium in an open access journal and make databases available in format similar to TCGA.
  - Coordinate biomarker validation.

## Objectives

Objectives of this consortium are outlined below.

**Fig. 2:**
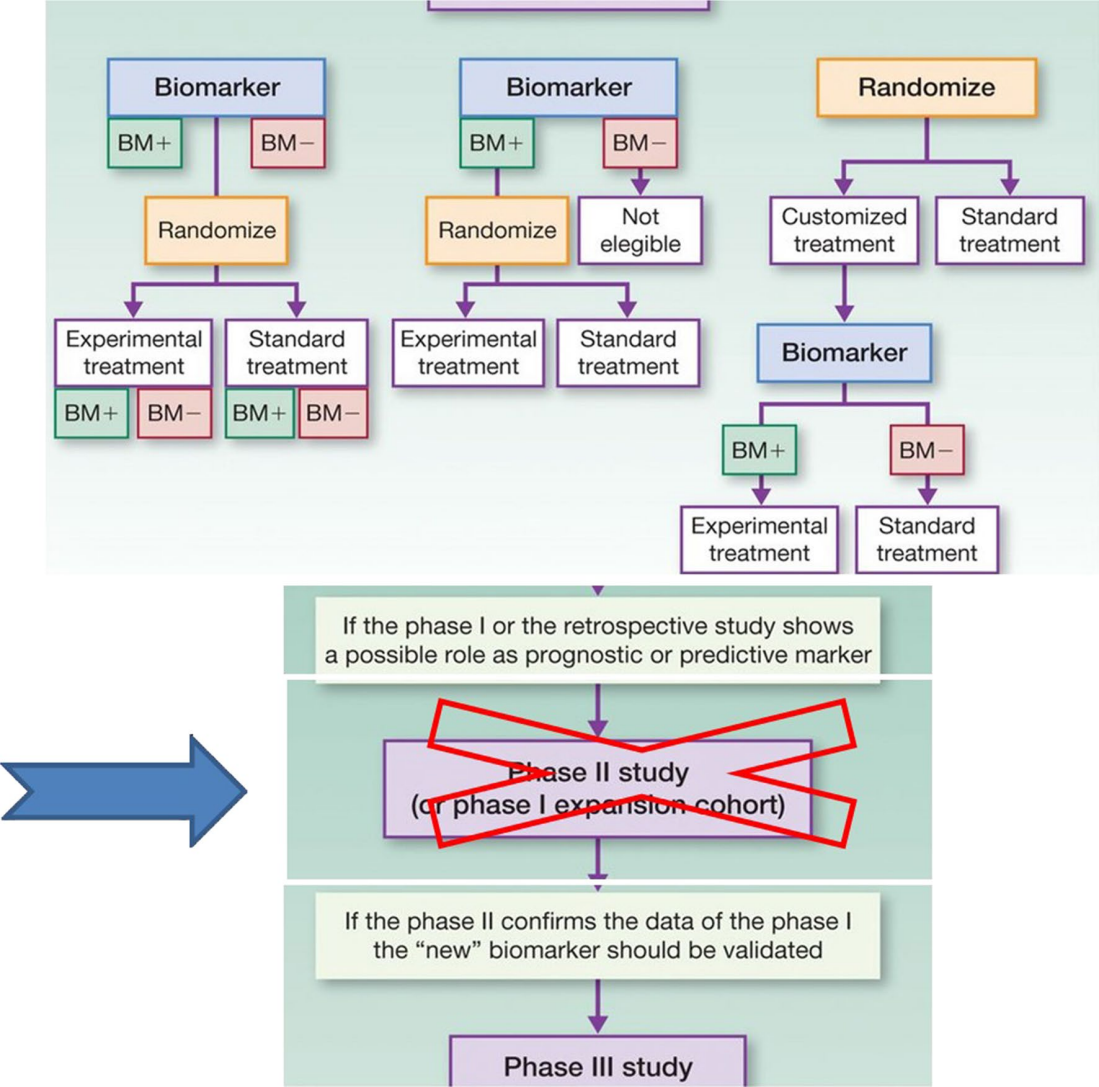
Reconstruction from Fig. 1
- A task force should be created with the intent to facilitate the discovery and development of predictive biomarkers.

- The creation of a database of available samples, with linked key information would be a top priority. This effort could start with the trial titles/therapeutics tested and basic trial design, number of patients, timepoints and the nature of biospecimens obtained, for a first review.
- Every participant would keep the samples on site but provide the information on this centralized database. An app that facilitates the collection of the data preserving the deidentified nature of the information is essential. This could also be designed to allow a relevant search engine to find samples based on specific characteristics.
- A committee would evaluate proposals after samples have been identified that may be suitable for a specific project.
- A network of bioinformaticians would be established with the purpose of developing new algorithms capable to interpret and integrate data coming from the analysis of different parameters/biomarkers (Fig. 2).

## Discussion

The main goal of this white paper is to develop the framework for a network of institutes with widespread sharing of available samples and clinical data. This will facilitate the exchange of knowledge and collaboration in suggesting upcoming clinical trials.

There are ongoing projects that could be partners such as the Foundation for the NIH Biomarker Consortium, in which projects are discussed among biomarker specialists, but then funded through collaborative industry funding in a ‘pre-competitive space’ where companion diagnostics are not yet ready to be developed by individual companies. In addition, the four existing working groups of the SITC Immunotherapy Biomarkers Task Force (http://sitc.sitcancer.org/about-sitc/initiatives) currently consist of a broad array of expertise in standardization and validation of biomarkers, new technologies, systematic high throughput approaches and baseline and tumor-focused biomarkers. In concert with these centralized steering committees, the involved academic centers that have clinical trials and individual biomarker expertise, would discuss what they have banked and what projects could be performed together.

Although it is relatively straightforward to create an Internal Cancer Immunotherapy Consortium, the major challenge is to execute the plan or methods of the network as described here. This requires stepwise planning and strategies based upon ‘what you have’ to ‘how you do’ to ‘what you could offer’ to ‘all we could achieve’. Both the central laboratory approach and alternative approach could start with retrospective biomarker analyses based upon samples which have been collected from immunotherapy trials from sites participating in the consortium, in order to highlight the quality of biomarker work from this consortium. The consortium could then work with pharmaceutical companies to launch potential prospective clinical trials to validate biomarkers. In addition, following the achievements of PD-L1 companion diagnostics for cancer immunotherapy, the consortium could initiate and facilitate efforts on transferring validated biomarkers such as RNA expression profile, tumor mutational burden to companion diagnostics. A clear, feasible and effective strategy will ensure the success of this consortium.

The concept of a network of collaborating biomarker discovery/validation efforts raises the critical question of potential mechanisms for financing this effort. Several initiatives are already emerging in this arena. In spring 2017 in the USA, the NCI initiated an effort that is supported with funds from the Beau Biden Cancer Moonshot Initiative (through peer review and NCI-support) for four separate laboratory centers to function as Clinical Immunotherapy Monitoring and Assessment Centers (CIMACs). However, the CIMAC laboratories network will serve NCI clinical trials in immunotherapy and would not have the capacity to integrate with other clinical trial networks/consortia. Perhaps in the future with more funding and expanded infrastructure this would be possible. These laboratory networks will engage in separate yet integrated laboratory activities using established and innovative platforms to evaluate both biomarkers and other surrogate endpoints for ongoing and future NCI sponsored cancer immunotherapy studies. This network could serve as an important model for correlative study infrastructure in other clinical trial networks and expanded interactions and coordination with other immune-oncology clinical laboratories for assessment and monitoring of biomarkers, at a global level.

The suggested international consortium for establishing and validating predictive biomarkers could participate in correlative biomarker science in clinical trials and also post-trial analyses in a systematic manner. This consortium could be very closely connected with clinical trials in immunotherapy including existing networks (e.g. the NCI supported Cancer Immunotherapy Trial Network, CITN) and all other ongoing trials in the US and Europe. Most trials will likely be small cohort studies. The consortium could combine several types of trials and analyze them across studies in an interdisciplinary approach. It is important to keep in mind possible bottlenecks. For instance, the US National Clinical Trials Network (NCTN, formerly the cooperative groups) has been attempting to develop a shared database across multiple specimen banks for some time and has found it to be a very complex task. Different institutes track different sample parameters and sharing any patient identifiers will be a concern with regard to privacy regulations. This is an issue that might be solved by applying similar parameters to categorize samples collected at each institution. Another example is the database for the pediatric brain cancer consortium, run at CHOP (Children’s Hospital of Philadelphia) using RedCap software with restricted consortium-only access [10]. While this is feasible, it requires expertise and personnel with dedicated effort.

In order to maintain solid communication and up-to-date information flow, the consortium could have monthly discussions and generate white papers summarizing promising data and outlining validation that could become high priority research initiatives to be taken on by the consortium or by other larger clinical trial groups with the capacity for such larger studies.

There are many important details that will require resolution in order to initiate a functional consortium able to pursue even a fraction of the objectives identified as important in this report. Moving forward will benefit from face-to-face discussions among a committed group of stakeholders, consisting of immune-oncology researchers with expertise in preclinical testing, clinical trials, and state-of-the-art laboratory evaluations of preclinical and clinical immunotherapy regimens. Convening a full-day workshop of such stakeholders, potentially at a SITC meeting, or a separate meeting of worldwide leaders in immune-oncology, may provide the time, focus, discussions, and interactions needed to help convert some of the helpful goals and concepts presented here into a prioritized action plan that might help promote this vision and enable additional next steps to proceed.
